# Weight Gain and Serum TSH Increase within the Reference Range after Hemithyroidectomy Indicate Lowered Thyroid Function

**DOI:** 10.1155/2014/892573

**Published:** 2014-05-14

**Authors:** Tina Toft Kristensen, Jacob Larsen, Palle Lyngsie Pedersen, Anne-Dorthe Feldthusen, Christina Ellervik, Søren Jelstrup, Jan Kvetny

**Affiliations:** ^1^Department of Otorhinolaryngology-Head and Neck Surgery, Koege Hospital, Region Zealand, 4600 Koege, Denmark; ^2^Department of Clinical Pathology, Naestved Hospital, Region Zealand, 4700 Naestved, Denmark; ^3^Department of Clinical Biochemistry, Naestved Hospital, Region Zealand, 4700 Naestved, Denmark; ^4^Department of Gynecology and Obstetrics, Naestved Hospital, Region Zealand, 4700 Naestved, Denmark; ^5^Department of Internal Medicine, Naestved Hospital, Region Zealand, 4700 Naestved, Denmark; ^6^University of Southern Denmark, 5230 Odense, Denmark

## Abstract

*Background*. Weight gain is frequently reported after hemithyroidectomy but the significance is recently discussed. Therefore, the aim of the study was to examine changes in body weight of hemithyroidectomized patients and to evaluate if TSH increase within the reference range could be related to weight gain. *Methods*. In a controlled follow-up study, two years after hemithyroidectomy for benign euthyroid goiter, postoperative TSH and body weight of 28 patients were compared to preoperative values and further compared to the results in 47 matched control persons, after a comparable follow-up period. *Results*. Two years after hemithyroidectomy, median serum TSH was increased over preoperative levels (1.23 versus 2.08 mIU/L, *P* < 0.01) and patients had gained weight (75.0 versus 77.3 kg, *P* = 0.02). Matched healthy controls had unchanged median serum TSH (1.70 versus 1.60 mIU/L, *P* = 0.13) and weight (69.3 versus 69.3 kg, *P* = 0.71). Patients on thyroxin treatment did not gain weight. TSH increase was significantly correlated with weight gain (*r* = 0.43, *P* < 0.01). *Conclusion*. Two years after hemithyroidectomy for benign euthyroid goiter, thyroid function is lowered within the laboratory reference range. Weight gain of patients who are biochemically euthyroid after hemithyroidectomy may be a clinical manifestation of a permanently decreased metabolic rate.

## 1. Background

Hemithyroidectomy is recommended as a treatment for a symptomatic unilateral benign nontoxic thyroid nodule or as a diagnostic surgical procedure in case of an indeterminate solitary thyroid nodule [1]. Whereas hypothyroidism is a recognized consequence of hemithyroidectomy with incidence rates ranging from 11% to 49% [2–4], only a single study has addressed the subject if lowered thyroid function within the laboratory reference range of the euthyroid majority of hemithyroidectomized patients may have clinical consequences [5]. It is the authors' experience that euthyroid patients, undergoing thyroidectomy, in spite of euthyroidism, report weight gain. However, studies concerning weight changes in euthyroid individuals after thyroidectomy are sparse. In 2011, it was reported that individuals after near total thyroidectomy, even if kept euthyroid by thyroxin supplement, gained more weight than euthyroid controls [6].

The aim of the present study was to examine if lowered metabolic rate, reflected by changes in TSH, could explain the weight gain, frequently reported by patients who remain within the euthyroid normal range of thyroid function after hemithyroidectomy for benign euthyroid goiter.

## 2. Materials and Methods

### 2.1. Hemithyroidectomy Group

This study was designed as a controlled follow-up study with a matched control group. The study group and control group have been described in detail previously [7]. We reviewed the hospital charts of all patients (*n* = 74) who underwent hemithyroidectomy in the time period from September 2008 to July 2009 at the Department of Otorhinolaryngology-Head and Neck Surgery, Slagelse Hospital, Hospital South, Region Zealand, Denmark. Criteria of inclusion were (1) no previous goiter surgery, (2) no previous radioiodine treatment for goiter, (3) no previous radiation therapy to the cervical region, (4) no previous or present medical treatment of hyperthyroidism or thyroid hormone substitution therapy, (5) preoperative value of TSH in the laboratory reference range (0.4–3.77 mIU/L), (6) benign pathologic diagnosis of resected tissue, (7) preoperative body mass index (BMI) between 20 and 35 kg/m^2^, and (8) age older than 18 years. Exclusion criteria were disease or medical treatment known to affect body weight.

Baseline (preoperative) measures of weight, height, and serum TSH were obtained from hospital charts. At the routine preoperative examination, patients' weight had been measured to the nearest 0.5 kg and height to the nearest 0.5 cm wearing indoor clothes and without shoes. A nonfasting venous blood sample had been drawn and analysed for serum concentrations of TSH.

Hemithyroidectomy was performed by one of four surgeons, who all used identical technique which involved total left or right thyroid lobectomy and isthmusectomy and, if present, resection of the pyramidal lobe with preservation of the contralateral thyroid lobe. The weight and morphology of the resected thyroid lobe were recorded.

Forty-six patients (11 men and 35 women) aged 32–74 years fulfilled the inclusion criteria and were invited to the study. Twenty-eight patients responded to the invitation (6 men and 22 women), resulting in a 61% participation rate. At the follow-up visit, patients reported medical history and current medication and were weighed to the nearest 0.5 kg wearing indoor clothes and without shoes, and a nonfasting venous blood sample was collected and analyzed for serum concentrations of TSH and antibodies against thyroid peroxidase (TPO-ab).

### 2.2. Control Group

The control persons were recruited from participants in the Danish General Suburban Population Study (GESUS) [8], which is conducted in the area of Denmark (with previous mild iodine deficiency) from which we also had recruited the hemithyroidectomy group. Control persons' criteria of inclusion were (1) reported absence of previous or present thyroid disease, (2) reported absence of previous or present medical treatment for thyroid disease, and (3) values of TSH and thyroid hormones within the laboratory reference ranges. Exclusion criteria were disease or medical treatment known to affect body weight. The control persons were matched for sex, age, BMI, smoking status, and presence of TPO-abs to the hemithyroidectomized patients. Ages were matched within 5-year age groups. BMI was matched to groups of BMI between 20 and 24.9, 25 and 29.9, and 30 and 35 kg/m^2^. Smoking status was matched to either current smoking status or no-smoking status, and presence of TPO-ab was matched to either TPO-ab positivity (TPO-ab > 60 kU/L) or TPO-ab negativity (TPO-ab < 60 kU/L). Baseline values of weight (to the nearest 0.5 kg), height (to the nearest 0.5 cm), and serum TSH were obtained from the GESUS database. Twenty months after the participation in the GESUS, we invited 80 controls (18 men, 62 women) to the study with the intention to include two controls for every hemithyroidectomized patient. Fifty-three persons responded to the invitation, resulting in a 66% participation rate. Six of these were excluded: two due to other ethnicity than Caucasian, two due to subsequent endocrine disease, and two due to antipsychotic medical treatment known to cause weight gain. Control persons reported medical history and current medication, they were weighed to the nearest 0.5 kg wearing indoor clothes and without shoes, and a nonfasting venous blood sample was collected and analyzed for serum concentrations of TSH. In total, the control group included 47 persons (9 men, 38 women). The heights obtained at the baseline examination of both study groups were used in the calculation of BMI at baseline and at follow-up. BMI was calculated as body weight (kg) divided by the square of the height (m).

### 2.3. Biochemical Variables

Measurements of TSH and of thyroid hormones free thyroxin (fT_4_) and total triiodothyronine (T_3_) were performed using an electrochemical luminescent immunoassay (Roche Cobas 6000, Basel, Switzerland). Reference range for TSH is 0.30–3.77 mIU/L (CV < 7%), fT_4_ is 10.0–26.0 pmol/L (CV < 5%), and T_3_ is 1.20–2.80 nmol/L (CV < 4%). Thyroid peroxidase antibody (TPO-ab) was measured by KRYPTOR anti-TPOn (BRAHMS, Hennigsdorf, Germany), with a detection limit of 10 kU/L.

### 2.4. Statistical Analysis

An a priori power calculation based on a TSH alteration of 0.1 mIU/L, alpha = 0.05, and power of 0.95, indicated a sample size of 27 patients in each group. Comparison between baseline values of responders and of nonresponders in both study groups was performed using the chi-square test for categorical variables, and as Shapiro-Wilk's test demonstrated absent normality of the measured parameters, the Mann-Whitney *U* test was used for continuous variables. Likewise, for the baseline comparison between the hemithyroidectomy and the control group, we used the chi-square test for categorical variables and the Mann-Whitney *U* test for continuous variables. The Wilcoxon signed-rank test was used to compare the change in medians of body weight and serum TSH of the hemithyroidectomy and the control group at baseline and at follow-up. The Spearman correlation coefficient was used to evaluate the correlation of the variables. Significant difference was defined as a *P* value < 0.05.

The match procedure and all analyses of results were evaluated using the STATA statistical package software programme (Statacorp, V. 12.0, College Station, TX, USA).

### 2.5. Ethical Considerations

The study was approved by the Regional Ethics Committee of Zealand, Denmark (SJ-10 and SJ-245), and by the Danish Data Protection Agency and registered in ClinicalTrials.gov (NCT01358136). The study conformed to the principles of the Declaration of Helsinki. Informed consent was obtained from all hemithyroidectomized patients prior to inclusion in the study. Informed consent was obtained from all control persons prior to participation in the GESUS as well as prior to inclusion in the current study.

## 3. Results

The indications of hemithyroidectomy (14 right and 14 left hemithyroidectomies) were suspicion of malignancy in 19 cases (67.9%) (cold areas in scintiscan or susceptible FNAB) and compressive symptoms in 9 cases (32.1%). The 28 hemithyroidectomized patients suffered no surgical complications such as secondary haemorrhage, infection, and temporary or permanent recurrent laryngeal nerve paresis. No patients were diagnosed histologically with Hashimoto's thyroiditis, although four patients showed increased TPO-ab > 60 kU/L (14.3%).

The participation rates were 61% in the hemithyroidectomy group and 66% in the control group.  Table 1 provides data that the nonresponders in both study groups did not differ from the responders regarding age, sex and baseline weight, BMI, or serum TSH.

Table 2 provides baseline data of the responders of the study groups, indicating that there were no statistically significant differences in sex, age, BMI, smoking status, and presence of TPO-abs. The baseline median TSH was significantly lower in the hemithyroidectomy group as previously reported [7] than in the control group (1.23, 0.91–1.51 versus 1.70, 1.10–2.20, *P* = 0.01), and the median duration of follow-up in the hemithyroidectomy group was significantly longer than in the control group (25, 23–30 versus 20, 19–21 months, *P* < 0.01).

Two years after hemithyroidectomy, 8 of 28 patients (28.6%) received thyroid hormone replacement therapy. Three patients (10.7%) demonstrated biochemical subclinical hypothyroidism with raised serum TSH values (>3.77 mIU/L) and values of fT_4_ and tT_3_ in the normal range, and they were referred to further evaluation by an endocrinologist. The remaining 17 patients (60.7%) were clinically euthyroid as was the case with those with subclinical hypothyroidism.

### 3.1. Postoperative Thyroid Function

**Figure 1:**
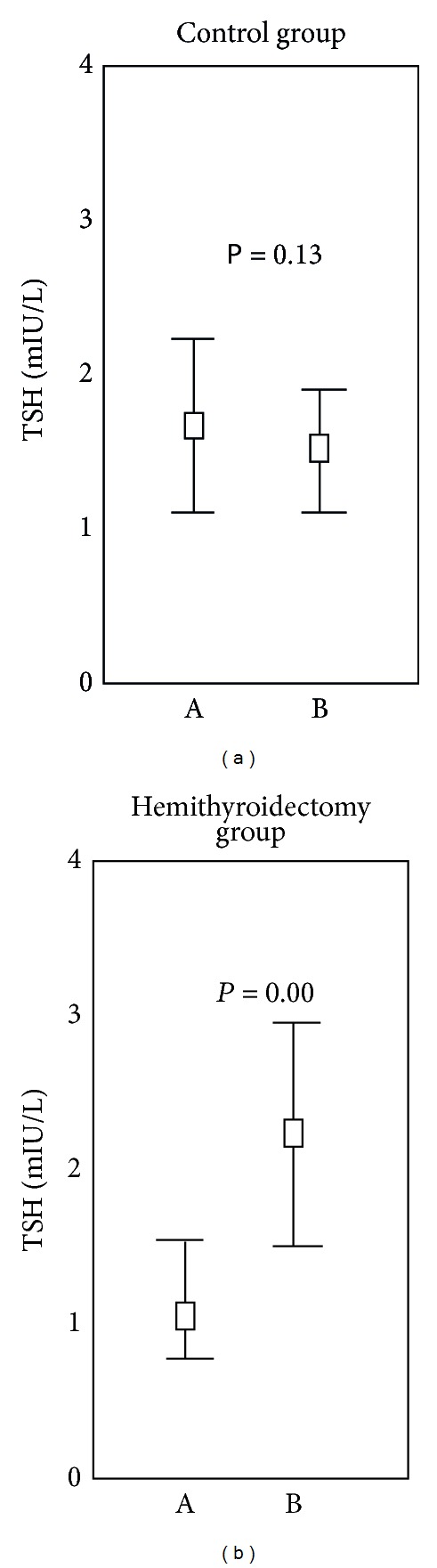
TSH changes between baseline and follow-up. TSH changes of the hemithyroidectomy group (*n* = 28) and of the control group (*n* = 47) after a two-year follow-up period. The median, upper, and lower quartiles are presented. Outliers are excluded. The Wilcoxon signed-rank test was used. Statistical significance was demonstrated whenever *P* < 0.05.

In total, 26 of the 28 patients (92.9%) showed increased levels of TSH over preoperative levels, compared to the control group in which 19 of 47 (40.4%) presented increased levels of TSH over baseline levels (*P* < 0.001).  Figure 1 presents the values of serum TSH of the hemithyroidectomy group and of the control group, measured at baseline and after follow-up. The figure demonstrates a significant TSH increase within the reference range of the hemithyroidectomy group two years after hemithyroidectomy, compared to the healthy control group.

### 3.2. Postoperative Body Weight

**Figure 2:**
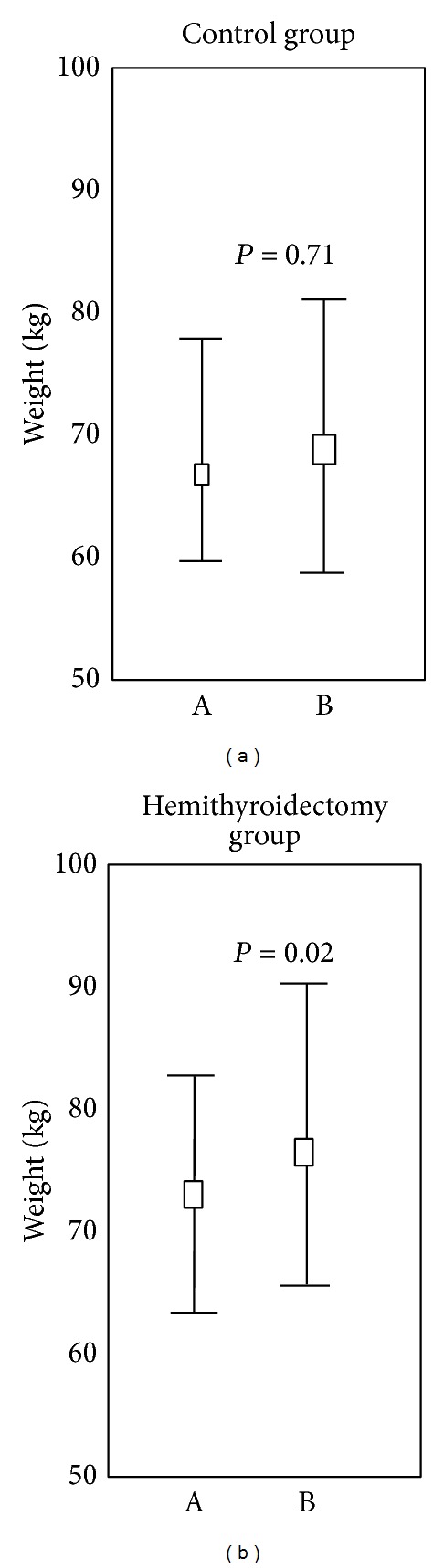
Weight changes between baseline and follow-up. Weight changes of the hemithyroidectomy group (*n* = 28) and of the control group (*n* = 47). The median, upper, and lower quartiles are presented. Outliers are excluded. The Wilcoxon signed-rank test was used. Statistical significance was demonstrated whenever *P* < 0.05.

Two years after hemithyroidectomy, 18 of 28 patients (64.3%) had gained weight and 10 of 28 had lost weight compared to the control group where 22 of 47 (46.8%) had gained weight and 25 of 47 had lost weight (*P* = 0.02).  Figure 2 presents the values of body weight of the hemithyroidectomy group and of the control group, measured at baseline and after follow-up. Two years postoperatively, the median body weight of the hemithyroidectomized group had increased significantly with an annual weight gain of 1.1 kg. In comparison, the median body weight of the healthy control group was unchanged after a 20-month follow-up.

In the 8 persons treated with thyroxin, the weight did not change. Weight before operation was 66 kg (59–76) versus weight after operation 66 kg (57–78, *P* = 0.75) (median and quartiles) in contrast to weight changes in the group without thyroxin treatment (Figure 2). Although the preoperative weight appears lower in the postoperative thyroxin treated group, the difference was not statistical significant (*P* = 0.06).

**Figure 3:**
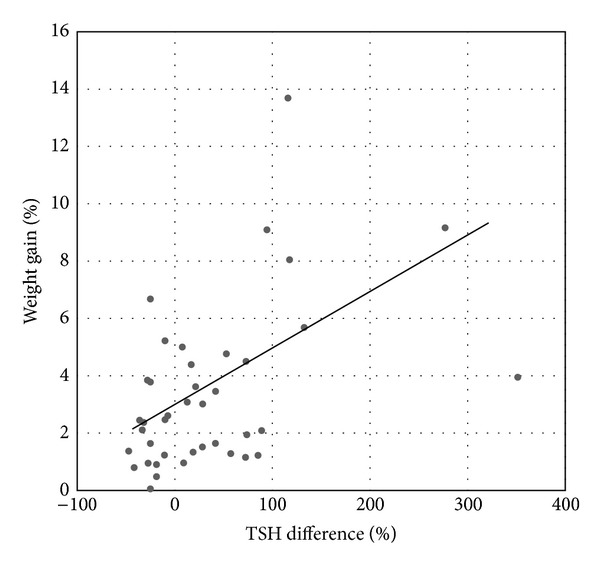
Correlation between relative changes in weight and serum TSH. Correlation between percent increase in weight and percent change in serum TSH of the hemithyroidectomy group (*n* = 18) and the control group (*n* = 22) during the follow-up period (*r* = 0.43, *P* < 0.01).

We examined the relation between changes in TSH and weight in the participants of both study groups, who had gained weight during follow-up, and we observed a significant correlation between the relative change in TSH and weight gain (Figure 3). We then examined the possibility of a factor to predict weight gain in the hemithyroidectomized group. Forward multiple logistic regression analysis with weight gain as dependent and preoperative weight, preoperative TSH, weight of resected thyroid tissue, and smoking status as independent variables did not, however, reveal a significant contributor.

## 4. Discussion

The major finding was a 1.1 kg annual weight increase in hemithyroidectomized person without thyroxin treatment compared to matched controls, who did not gain weight. Our result regarding postoperative TSH is in coherence with previous studies' reports of increased TSH within the laboratory reference range 1 and 3 years after hemithyroidectomy [2, 5, 9, 10]. The clinical significance of a posthemithyroidectomy TSH increase has not, however, previously been addressed. In the present study, 28.6% received levothyroxine two years after hemithyroidectomy, which is comparable to the posthemithyroidectomy hypothyroidism rates reported in the literature [2–4] and it is noteworthy that these patients did not gain weight. The exact clinical consequences of having a permanently increased TSH after hemithyroidectomy still need to be studied.

Serum TSH levels within the laboratory reference range have been associated with BMI in cross-sectional population-based studies [11] and, in a longitudinal design, increase in TSH over time, but within the euthyroid range they were associated with weight gain [12, 13]. Moreover, it has been demonstrated that small variations in thyroid function, with serum TSH variations within the normal laboratory range, resulted in decreased resting energy expenditure of patients on T_4_ substitution [14]. These studies indicate that the well-known association between low thyroid function and weight gain extends into the euthyroid states and propose the hypothesis that an increase in TSH reflects decreased metabolic rate causing weight gain and not the other way around [15, 16]. In support of this hypothesis speaks the present study, where TSH increase, induced by hemithyroidectomy for benign unilateral goiter, is significantly related to weight gain and the fact that thyroxine treated patients did not gain weight. Weight loss prior to operation may have contributed as a possible malignancy would cause concern in some and hence induce weight loss.

The pituitary gland is sensitive to small changes in serums T_4_ and T_3_ and, before and after thyroid surgery, measurement of serum TSH is used routinely to screen for thyroid dysfunction. Each individual has a unique level of thyroid function, and the individual normal ranges for thyroid function are much narrower than the population-based reference ranges [17]. Postoperative test results that are abnormal for the individual can therefore be unnoticed in the wide population-based reference range if the preoperative thyroid function is not taken into consideration. In this setting, the 1.1 kg annual weight gain of the hemithyroidectomy group might be a clinical reflection of a permanently increased TSH to a level that is outside the normal range of the individual.

The annual weight gain of 1.1 kg of the hemithyroidectomy group is relatively modest. However, it is still concerning and could over time become clinically significant. In a previous study, total thyroidectomy for hyperthyroidism resulted in continuous weight gain during a 4-year follow-up period [18], suggesting that weight gain after thyroid surgery is more likely a reflection of a permanently changed thyroid function than it is a regain of previously lost weight. We did not find a factor that could predict weight gain after hemithyroidectomy, which might be explained by the limited number of participating patients.

The study is limited by its follow-up design and the 61% participation rate, although this rate is comparable to (and even in the higher spectrum of) that reported in population studies. Reasons for nonresponse may be due to long distance to the outpatient clinic and the fact that the hemithyroidectomized patients are generally healthy and employed. The major concern with the participation rates of the study group is the potential for nonresponse bias. However, the nonresponding hemithyroidectomized patients and controls did not differ from the responding ones regarding preoperative weight, BMI, or thyroid function (Table 1), and we find it unlikely that postoperative and follow-up thyroid function and body weight of nonresponders should be different from the responders'. Another limitation is the heterogeneity of collection of preoperative body weight data of the hemithyroidectomy group, since they have been weighed by different health care providers at the routine preoperative examination. The follow-up study design may have had an advantage since engagement in life style changes of the hemithyroidectomy group due to higher awareness of the risk of weight gain could not have had any confounding effect on the results and it is important to notice that the 1.1 kg annual weight gain of the hemithyroidectomy group is considerably greater than the reported annual weight increase of 0.4 kg of westernized populations [19].

## 5. Conclusion

Two years postoperatively, serum TSH of hemithyroidectomized patients was significantly increased within the reference range and patients had gained weight significantly. Our study confirms the patient-reported weight gain after hemithyroidectomy and proposes the hypothesis that weight gain of patients who are biochemically euthyroid after hemithyroidectomy for benign euthyroid goiter may be a clinical manifestation of a permanently decreased metabolic rate.

## Figures and Tables

**Table 1:** Comparison between responders and nonresponders of the study groups.

Responders	Control group			Hemithyroidectomy group		
	Yes (*n* = 47)	No (*n* = 27)	*P* value	Yes (*n* = 28)	No (*n* = 18)	*P* value
Males (%)	19.1	33.3	0.17*	21.4	50.0	0.37*
Age (years)	53 (43–62)	47 (40–58)	0.22	51.5 (43–60)	53.5 (45–58)	0.81
Weight (kg)	69.3 (62.2–81.4)	78.5 (67.5–92.0)	0.16	75.0 (63.5–85.0)	87.0 (65.0–98.0)	0.87
BMI (kg/m^2^)	25.0 (22.7–29.0)	27.7 (23.6–29.9)	0.24	26.1 (23.1–28.3)	29.1 (23.9–32.3)	0.17
TSH (mIU/L)	1.70 (1.10–2.20)	1.70 (1.10–2.20)	0.85	1.23 (0.91–1.59)	1.02 (0.68–1.57)	0.17

The Mann-Whitney *U* test for continuous variables (median and interquartile range are presented). *Chi-square test was for categorical variables. Significant difference was defined as a *P* value < 0.05. BMI: body mass index; TSH: thyroid stimulating hormone.

**Table 2:** Comparison between study groups at baseline.

Variables	Control group (*n* = 47)	Hemithyroidectomy group (*n* = 28)	*P* value
Males (%)	19.2%	21.4%	0.81*
Age (years)	53 (43–62)	51.5 (43–60)	0.59
Weight (kg)	69.3 (62.2–81.4)	75.0 (63.5–85.0)	0.43
BMI (kg/m^2^)	25.0 (22.7–29.0)	26.1 (23.1–28.3)	0.65
Smokers (%)	8.5	14.3	0.43*
TPO-ab positivity (%)	12.8	14.3	0.85*

The Mann-Whitney *U* test was used for continuous variables (median and interquartile range are presented). *Chi-square test for categorical variables. Significant difference was defined as a *P* value < 0.05. BMI: body mass index; TPO-ab: thyroid peroxidase antibody.
